# Structural insight into proline *cis*/*trans* isomerization of unfolded proteins catalyzed by the trigger factor chaperone

**DOI:** 10.1074/jbc.RA118.003579

**Published:** 2018-08-09

**Authors:** Soichiro Kawagoe, Hiroshi Nakagawa, Hiroyuki Kumeta, Koichiro Ishimori, Tomohide Saio

**Affiliations:** From the ‡ Graduate School of Chemical Sciences and Engineering, Hokkaido University, Sapporo, Hokkaido 060-8628, Japan,; the § Materials Sciences Research Center, Japan Atomic Energy Agency, Tokai, Ibaraki 319-1195, Japan,; the ¶ Faculty of Advanced Life Science, Hokkaido University, Sapporo, Hokkaido 011-0021, Japan,; the ‖ Department of Chemistry, Faculty of Science, Hokkaido University, Sapporo, Hokkaido 060-0810, Japan, and; ** PRESTO, Japan Science and Technology Agency, Tokyo 102-0076, Japan

**Keywords:** molecular chaperone, prolyl isomerase, molecular dynamics, protein folding, structure-function, nuclear magnetic resonance (NMR), hydrophobic interaction, peptidyl-prolyl isomerase domain, trigger factor

## Abstract

Molecular chaperones often possess functional modules that are specialized in assisting the formation of specific structural elements, such as a disulfide bridges and peptidyl–prolyl bonds in *cis* form, in the client protein. A ribosome-associated molecular chaperone trigger factor (TF), which has a peptidyl–prolyl *cis*/*trans* isomerase (PPIase) domain, acts as a highly efficient catalyst in the folding process limited by peptidyl–prolyl isomerization. Herein we report a study on the mechanism through which TF recognizes the proline residue in the unfolded client protein during the *cis*/*trans* isomerization process. The solution structure of TF in complex with the client protein showed that TF recognizes the proline-aromatic motif located in the hydrophobic stretch of the unfolded client protein through its conserved hydrophobic cleft, which suggests that TF preferentially accelerates the isomerization of the peptidyl–prolyl bond that is eventually folded into the core of the protein in its native fold. Molecular dynamics simulation revealed that TF exploits the backbone amide group of Ile^195^ to form an intermolecular hydrogen bond with the carbonyl oxygen of the amino acid residue preceding the proline residue at the transition state, which presumably stabilizes the transition state and thus accelerates the isomerization. The importance of such intermolecular hydrogen-bond formation during the catalysis was further corroborated by the activity assay and NMR relaxation analysis.

## Introduction

Newly synthesized proteins, nascent polypeptides emerge out of the ribosome in an unstructured nonnative state and are then folded into their innate three-dimensional structures to fulfill their specific biological activities (1). Protein folding often requires the assistance of molecular chaperones. The molecular chaperones not only transiently protect the client proteins from the nonspecific hydrophobic interactions with other proteins (2, 3) but also assist in the formation of specific structural elements in the client proteins, such as disulfide bridges or peptidyl–prolyl bonds in *cis* form that are often required in the native fold. Because the formation of these specific structural elements can be a rate-limiting step in the folding pathway, several molecular chaperones possess specific functional modules that are specialized for the formation of such structural elements (4–7).

Trigger factor (TF),^3^ a bacterial chaperone that interacts with nascent polypeptides immediately after their emergence from the ribosome (8), has an FK506-binding protein (FKBP)-type peptidyl–prolyl *cis*/*trans* isomerase (PPIase) domain (PPD), along with the other two domains, *i.e.* the ribosome-binding domain (RBD) and the substrate-binding domain (SBD) (9). The transition between the *cis* and *trans* forms of peptidyl-prolyl bond, in which the ω dihedral angle is ∼0° or 180°, respectively, occurs very slowly in the absence of PPIases because of the high energy barrier imposed by the partial double bond character of the peptide bond. Even though the *trans* form is usually more populated than the *cis* form in the unfolded proteins, the protein folding may require either of the two forms to fit in the native fold. TF acts as a highly efficient catalyst in the folding process limited by peptidyl–prolyl isomerization (10, 11). Consequently, deletion of the TF gene results in severe aggregation of many proteins including cytosolic and membrane proteins as well as the cold-sensitive phenotype (12, 13). Even *Mycoplasma genitalium*, which comprises a minimal set of genes, has TF as a sole PPIase (14), highlighting the importance of the PPIase activity of TF in protein biogenesis.

Despite its important roles in the cell, the mechanism through which TF^PPD^ catalyzes the peptidyl–prolyl *cis*/*trans* isomerization, as well as the mechanism for the other members of the FKBP-type PPIase family, still remains unclear. Although PPIases are thought to promote proline *cis*/*trans* isomerization by stabilizing the high energy transition state, the mechanism of this stabilization has not been clarified to date, even after several important works including biochemical studies on TF^PPD^ (15, 16) and crystallographic studies on *Pv*FKBP35 (17) and SlyD (18, 19) in complex with proline-containing peptides. The lack of a comprehensive structural study that can track the entire *cis*/*trans* isomerization process has hindered the elucidation of the mechanism. Further insight into the structure of the PPIase in complex with the client protein both at the ground state and at the transition state is required.

Herein we report an integrated study on TF^PPD^ exploiting solution NMR, molecular dynamics (MD) simulation, and activity assay. The solution structure of TF^PPD^ in complex with a proline-containing fragment of maltose-binding protein (MBP) has unveiled the mechanism by which the proline residue is recognized by TF^PPD^, and MD simulation has shed light on the complex at the transition state. Our results show that the conserved hydrophobic cleft of TF^PPD^ recognizes the *trans* form of the proline-aromatic motif in the client protein and that an intermolecular hydrogen bond between the backbone amide group of Ile^195^ in TF^PPD^ and the carbonyl oxygen of the amino acid residue preceding the proline residue in MBP is formed at the transition state. These results suggest that the hydrophobic environment around the peptidyl–prolyl bond and the intermolecular hydrogen bond at the transition state play major roles in the proline *cis*/*trans* isomerization.

## Results

### TF^PPD^ recognizes proline residue in the hydrophobic region

**Figure 1.**
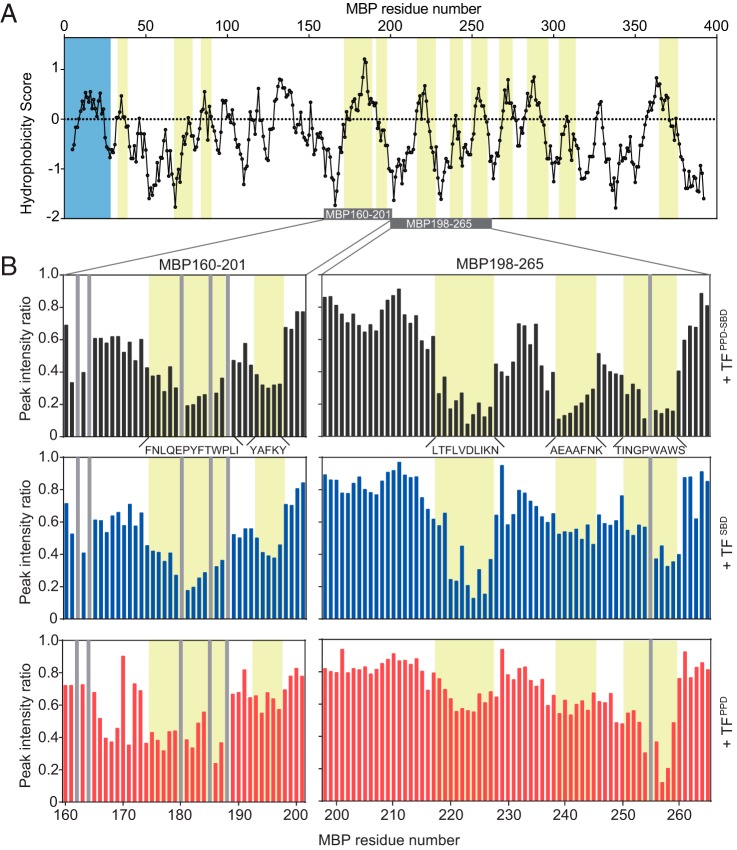
**Recognition of unfolded MBP by TF.** *A*, plot of the hydrophobicity score (Roseman algorithm, window = 9) of MBP as a function of its primary sequence. A hydrophobicity score higher than 0 denotes increased hydrophobicity. The regions recognized by TF as identified by NMR titration experiments are highlighted in *yellow*. The signal sequence is highlighted in *blue*. The MBP segments depicted in the *B* are indicated as a *gray bar. B*, plots of peak intensity change of MBP160–201 (*left panels*) and MBP198–265 (*right panels*) by the addition of TF^PPD-SBD^ at a ratio of MBP:TF^PPD-SBD^ 1:0.1 (*top panels*), TF^SBD^ at a ratio of MBP:TF^SBD^ 1:0.2 (*middle panels*), and TF^PPD^ at a ratio of MBP:TF^PPD^ 1:0.5 (*bottom panels*), as a function of the primary sequences of the MBP peptides. The regions recognized by TF are highlighted in *yellow* and labeled with the primary sequences. The proline residue and unassigned residue are indicated by *gray bar*.

Interaction between TF (Fig. S1*A*) and an unfolded substrate protein was first investigated by NMR. MBP was used as an unfolded substrate protein. To ensure the solubility and stability in the unfolded state, MBP was divided into short fragments and six fragments of MBP (20) were prepared: MBP29–99, MBP97–164, MBP160–201, MBP198–265, MBP260–336, and MBP331–396. All of the MBP fragments exhibited narrow chemical shift dispersion in the ^1^H-^15^N HSQC spectra, which is characteristic to unfolded proteins (Fig. S1*B*). MBP consists of 396 amino acids and contains 21 proline residues. To identify the interaction sites on the unfolded MBP, the tandem domain TF^PPD-SBD^, which possesses all of the five substrate-binding sites (21), was titrated into isotopically labeled MBP fragments, and the perturbation of each resonance from the MBP fragments was monitored (Fig. S1*B*). The addition of TF^PPD-SBD^ induced a significant reduction in the intensity of the resonances because of the size of the protein and the binding kinetics. Differential line broadening analysis showed that a total of 12 regions of MBP are recognized by TF (Fig. 1*A* and Fig. S2*A*). The continuous stretch consisting of more than four amino acid residues with significant intensity reduction upon the addition of TF^PPD-SBD^ was defined as a binding site. These TF-recognition sites contain highly hydrophobic regions (Fig. 1*A*) and are located on the core of MBP in its native fold (Fig. S2*B*). The regions recognized by TF are enriched with hydrophobic amino acids including aromatic residues, such as tryptophan and phenylalanine, as well as bulky hydrophobic aliphatic amino acids such as leucine and valine (Fig. S2, *C* and *D*). This binding preference of TF toward hydrophobic and aromatic amino acid residues is consistent with the results obtained in the previous studies that used other unfolded protein substrates (21, 22). The regions recognized by TF closely resemble those recognized by SecB chaperone (20), which suggests that the two chaperones share similar binding specificity. Thermodynamics parameters of the interaction between TF^PPD-SBD^ and MBP198–265 estimated by isothermal titration calorimetry (ITC) experiment (Fig. S3), Δ*G* −5.8 ± 0.1 kcal/mol, Δ*H* −5.9 ± 0.4 kcal/mol, and −*T*Δ*S* 0.1 ± 0.5 kcal/mol, indicated that the binding is enthalpy-driven. The same trend was also seen for another bacterial chaperone SecB interacting with unfolded MBP (20), implying the shared binding properties between the two chaperones. The dissociation constant *K_d_* was calculated as 47 ± 9 μm, indicating that the binding between TF and MBP is relatively weak. Among the 21 proline residues in the MBP sequence, four residues are located in the hydrophobic regions and are recognized by TF (Fig. S2, *A* and *D*). The proline residues in the hydrophilic regions did not interact with TF, which is in accord with the previous report showing that TF has no specificity toward proline residue (22).

To further investigate the recognition of the proline-containing hydrophobic stretches of MBP by TF, the two fragments of MBP containing proline residues in the hydrophobic stretches, MBP160–201 and MBP198–265, were subjected to titration experiments with isolated domains of TF, TF^PPD^ and TF^SBD^. Both domains were titrated into isotopically labeled MBP160–201 and MBP198–265, and the interaction was monitored by NMR (Fig. S4*A*). Several resonances on the ^1^H-^15^N HSQC spectra were specifically affected by the addition of TF^PPD^ or TF^SBD^. A significant line broadening, with a consequent intensity reduction, was observed by the addition of TF^SBD^, whereas both line broadening and chemical shift perturbation were observed upon the addition of TF^PPD^ (Fig. S4*A*), reflecting the difference between the domains with regard to binding kinetics and molecular weight. For clarity, intensity changes were evaluated and compared in the analysis. As seen in the titration of TF^PPD-SBD^, the addition of TF^SBD^ and TF^PPD^ induced a significant intensity reduction for specific resonances of the MBP160–201 and MBP198–265 (Fig. 1*B* and Fig. S4*A*). These resonances are from the following MBP regions: Phe^175^–Ile^187^, Tyr^193^–Tyr^197^, Leu^218^–Asn^227^, Ala^239^–Lys^245^, and Thr^251^–Ser^259^. All of these five binding sites are affected by the addition of isolated domains of TF^PPD^ and TF^SBD^. However, the extent of peak intensity change varies among the binding sites: in the titration experiments for isotopically labeled MBP198–265, the most significant peak intensity reductions were observed for the resonances attributed to the MBP stretch Thr^251^–Ser^259^ upon the addition of TF^PPD^, whereas the addition of TF^SBD^ caused the most significant effects on the resonances from the MBP stretch Leu^218^–Asn^227^ (Fig. 1*B*). Given the relatively weak affinity between the MBP fragment and TF as expected from ITC data (Fig. S3) and the fact that the peak intensity change was monitored by the addition of substoichiometric amount of TF proteins, more significant intensity reduction, thus more significant line broadening, is attributed to slower binding kinetics, which tends to occur in stronger interaction (23) and/or higher population of the bound form. In either case, the extent of the peak broadening can be interpreted as an indication of the binding preference. Thus the data suggest that the MBP stretches Leu^218^–Asn^227^ and Thr^251^–Ser^259^ are preferred by TF^SBD^ and TF^PPD^, respectively. Note that the plots of chemical shift change by the addition of TF^PPD^ showed the same trend (Fig. S4*B*), further supporting the preference of TF^PPD^. Interestingly, the hydrophobic stretch of Thr^251^–Ser^259^ contains two tryptophan residues and one proline residue, whereas the other two stretches in MBP198–265 are devoid of the pair of tryptophan and proline residues. This result suggests that TF^PPD^ possesses a preference toward aromatic and proline residues located in the hydrophobic regions. Although the previous biochemical study found no sequence specificity of TF toward proline residues (22), the effectiveness of the NMR technique in the analysis of weak interactions enabled us to discover the weak preference.

**Figure 2.**
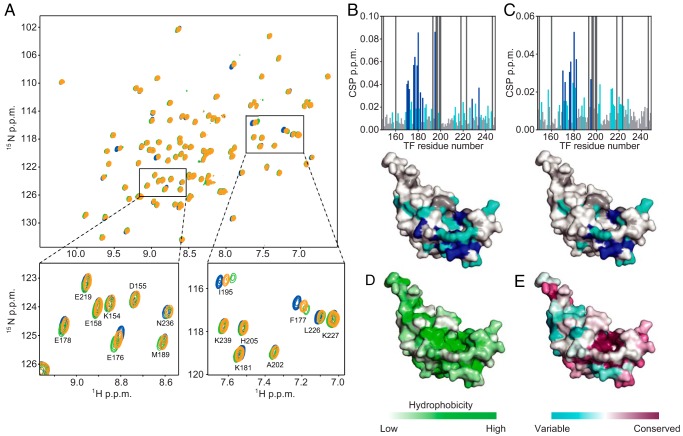
**The substrate-binding sites on TF^PPD^.** *A*, ^1^H-^15^N HSQC spectra of TF^PPD^ in the absence (*blue*) and presence of MBP160–201 (*green*) or MBP198–265 (*orange*). The spectra were recorded with several titration points, TF^PPD^:MBP 1:0.1, 1:0.2, 1:0.5, 1:1, and 1:2, but only the spectra at 1:2 ratio are shown for clarity. The regions indicated by *boxes* in the *top panel* are expanded in the *bottom panels* with resonance assignments. *B* and *C*, chemical shift perturbations of amide moieties of TF^PPD^ upon binding to MBP160–201 (*B*) or MBP198–265 (*C*), plotted as a function of the residue number of TF^PPD^. Proline and unassigned residues are indicated by *bars* colored *dark gray*. The *lower panels* show chemical shift perturbation mapping on the structure of TF^PPD^ (PDB code 1W26). The residues with perturbations larger than the threshold values of 0.015 and 0.025 ppm are colored *light blue* and *dark blue*, respectively. Proline and unassigned residues are colored *dark gray. D*, mapping of the hydrophobicity on the structure of TF^PPD^. *E*, TF^PPD^ sequence conservation mapped on the structure of TF^PPD^.

To identify the interaction site on TF^PPD^, we further investigated the interaction by NMR titration experiments observing the resonances from TF^PPD^. MBP160–201 or MBP198–265 was titrated into isotopically labeled TF^PPD^ (Fig. 2*A*). The addition of the MBP fragment induced significant chemical shift perturbations for the resonances of TF^PPD^ (Fig. 2, *B* and *C*). The chemical shift perturbation mapping shows that the majority of the perturbed resonances are from the conserved hydrophobic surface of TF^PPD^ (Fig. 2, *B–E*). These chemical shift changes are in the fast exchange regime (Fig. 2*A* and Fig. S5), which indicates a highly dynamic binding mode between TF^PPD^ and unfolded MBP, as was previously reported for unfolded PhoA (21). Relatively small chemical shift perturbations indicate the moderate affinity between the isolated TF^PPD^ and MBP. The data indicate that the interaction with the substrate protein becomes weaker in the absence of TF^SBD^ baring four substrate-binding sites, as also shown in the previous report (21).

### Structural basis for the recognition of proline residue in the substrate protein

**Figure 3.**
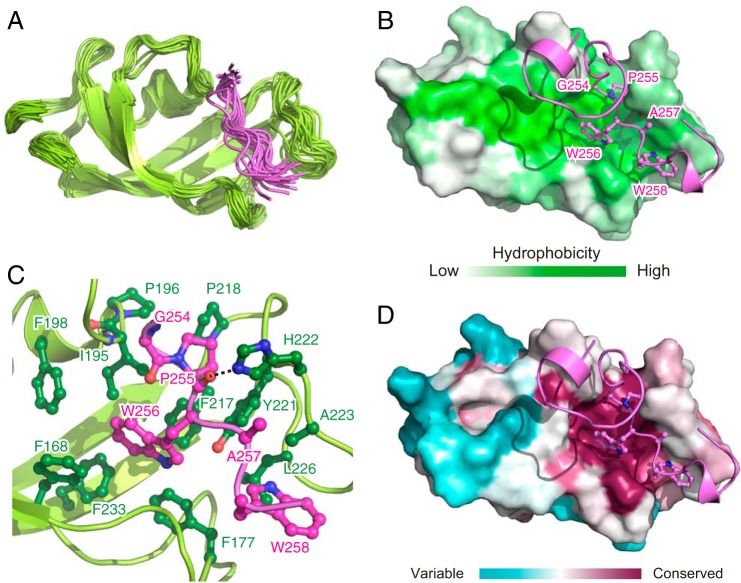
**NMR structure of TF^PPD^ in complex with MBP.** *A*, superimposition of 20 structures of TF^PPD^ in complex with MBP 238–266. For MBP, only the converged region, Asn^253^–Ser^259^, is shown for clarity. TF^PPD^ and MBP are shown in *green* and *pink ribbons*, respectively. *B* and *D*, the lowest-energy structure of MBP–TF^PPD^ complex. TF^PPD^ is shown as a solvent-exposed surface to which the hydrophobicity (*B*) or the conservation of the amino acid residues (*D*) is mapped. MBP is shown as *pink ribbons*. The MBP residues that directly interact with TF^PPD^ are drawn in a ball-and-stick representation. *C*, close-up view of the lowest-energy structure of MBP–TF^PPD^ complex. TF^PPD^ and MBP are shown as *green* and *pink ribbons*, respectively. The residues of TF^PPD^ and MBP involved in the interaction are drawn in a ball-and-stick representation and colored *green* and *pink*, respectively.

To gain a better insight into the recognition of the MBP stretch containing proline and aromatic residues by TF^PPD^, we conducted a structural analysis of TF^PPD^ in complex with unfolded MBP, focusing on the MBP stretch Thr^251^–Ser^259^ that contains both proline and aromatic residues. Because the relatively low affinity between TF^PPD^ and MBP results in a low population of the complex when the two proteins are mixed in solution, a fusion protein was designed to increase the population of the bound state, which would increase the number of intermolecular NOEs for high-resolution structure determination. To this aim, the peptide containing the binding site (MBP238–266) was fused to the N terminus of TF^PPD^ with a linker consisting of five repeat units of Gly-Ser. The directions of the chemical shift perturbations caused by the fusion of the MBP peptide coincided with those resulting from the addition of the isolated MBP fragment (Fig. S5), indicating that the interaction between TF^PPD^ and MBP238–266 is preserved in the fusion protein. Moreover, much more significant chemical shift perturbations were observed for the fusion protein, which indicates that a much higher population of the MBP peptide is bound to TF^PPD^ in the fusion. The solution structure of MBP–TF^PPD^ complex was determined on the basis of 1205 NOE-derived interproton distance restraints, 128 dihedral angle restraints, and 20 hydrogen-bond restraints (Table S1). A total of 100 structures were calculated, among which the 20 lowest energy structures were selected (Fig. 3*A*).

The structure showed that TF^PPD^ recognizes the hydrophobic stretch of MBP, Pro^255^–Trp^258^, using the broad hydrophobic surface (Fig. 3*B*) that is also used for the recognition of the other unfolded substrate proteins including PhoA and OmpA (21). The indole ring of MBP Trp^256^ inserts into the hydrophobic pocket of TF^PPD^ formed by Phe^168^, Phe^177^, Ile^195^, Phe^198^, Phe^217^, Tyr^221^, and Phe^233^ (Fig. 3*C* and Fig. S6*A*). MBP Pro^255^, which is located next to the tryptophan residue, is captured by the hydrophobic cleft formed by Ile^195^, Pro^196^, Phe^217^, Pro^218^, Tyr^221^, and His^222^. The amino acid residues of TF^PPD^ that are involved in the recognition of the sequence MBP Pro^255^–Trp^256^ are highly conserved (Fig. 3*D*). Interestingly the proline-aromatic amino acid sequence is also found in the other peptide sequence recognized by TF^PPD^. For example, MBP Pro^180^, which is located next to tyrosine residue (Tyr^181^), was found to be recognized by TF^PPD^ from NMR titration experiments (Fig. 1*B* and Fig. S6*B*), and RNase T1 Pro^39^ and Pro^55^, whose *cis*/*trans* isomerization is known to be catalyzed by TF^PPD^ (15), are located next to tyrosine or histidine residues (Fig. S6*B*). Taking these observations into consideration, we propose the proline-aromatic motif as the target for the recognition and *cis*/*trans* isomerization by TF^PPD^. In addition to MBP Pro^255^–Trp^256^, another tryptophan residue in the hydrophobic stretch, Trp^258^, is recognized by TF^PPD^ through the interaction with the hydrophobic cleft formed by Phe^177^, Tyr^221^, Ala^223^, and Leu^226^ (Fig. 3*C*). In addition to the hydrophobic interactions, a hydrogen bond between TF^PPD^ His^222^ N^ϵ2^ and the backbone carbonyl oxygen of MBP Pro^255^ is found in the complex. All these interactions contribute to holding tightly the MBP stretch Pro^255^–Trp^258^. On the other hand, MBP Gly^254^, which is located at the N terminus of Pro^255^, is positioned at the edge of TF^PPD^, and no significant contact with TF^PPD^ was observed. It is worth noting that in the structure of the MBP–TF^PPD^ complex, in which Pro^255^ is in the *trans* form, the backbone carbonyl oxygen of MBP Gly^254^ is located nearby the backbone amide proton of TF^PPD^ Ile^195^, but not close enough to form a hydrogen bond (Fig. 3*C*).

Although MBP Pro^255^ in the unbound MBP198–265 is in slow exchange between *trans* and *cis* forms as represented by the two sets of the NMR signals (Fig. S6*C*), MBP Pro^255^ in complex with TF^PPD^ was found to adopt the *trans* form, as can be deduced from the single set of the resonances whose chemical shifts are in the range expected for *trans* form (24) (Fig. S6*D*). This observation was consistent with the results of the titration experiments in which the resonances from MBP were monitored (Fig. S6*E*). When TF^PPD^ was titrated into isotopically labeled MBP160–201 or MBP198–265, the resonances corresponding to the *trans* form showed more significant peak intensity reduction (Fig. S6*E*, *lower panels*), implying stronger interaction between TF^PPD^ and the *trans* form of the substrate protein. On the other hand, this trend was not observed upon the addition of TF^SBD^ (Fig. S6*E*, *upper panels*). These results indicate the binding preference of TF^PPD^ toward proline residue in the *trans* form.

### An intermolecular hydrogen bond is formed at the transition state in the cis/trans isomerization

**Figure 4.**
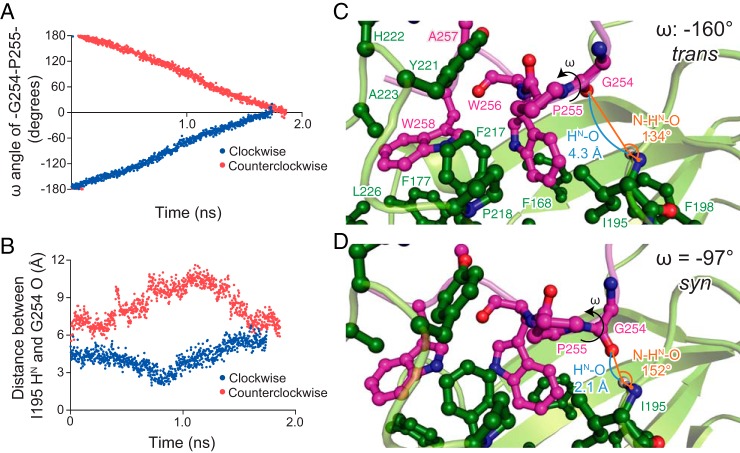
**Molecular dynamics simulation of *cis*/*trans* isomerization by TF^PPD^.** *A*, plots of the ω dihedral angle for the peptidyl–prolyl bond between MBP Gly^254^ and Pro^255^. The simulation was started from the NMR structure of MBP–TF^PPD^ complex in which the ω angle for the peptidyl–prolyl bond between MBP Gly^254^ and Pro^255^ is −174° (*trans* state). Because of the ω angle constraint at a rate of 0.2° per 2 ps, the ω angle changed linearly as a function of time. *B*, plots of the distance between the H^N^ atom of TF Ile^195^ and the carbonyl oxygen of MBP Gly^254^ as a function of time when the ω angle was rotated clockwise (*blue*) or counterclockwise (*red*). When the ω angle was rotated in clockwise direction, the two atoms approached each other, and the distance became shorter than 2.5 Å at ∼0.8 ns. As seen in *A*, the ω dihedral angle at 0.8 ns was at approximately −90° (*syn* state). *C* and *D*, snap shots of the *cis*/*trans* isomerization at the *trans* state (0.200 ns) (*C*) and the *syn* state (0.794 ns) (*D*). The intermolecular hydrogen bond between backbone amide group of TF^PPD^ Ile^195^ and the backbone carbonyl oxygen of MBP Gly^254^ is formed at the *syn* state, which tethers the peptidyl–prolyl bond closer onto the hydrophobic cleft formed by TF^PPD^ Ile^195^, Phe^217^, and Pro^218^.

To investigate the recognition of the substrate protein by TF^PPD^ during the *cis*/*trans* transition, we performed MD simulation, using the lowest-energy structure of the MBP–TF^PPD^ complex determined by NMR as a starting point. Because proline *cis*/*trans* isomerization is slow compared with the timescale of MD simulation, a constrained MD simulation was performed in which a rotational angle constraint for the ω angle between MBP Gly^254^ and Pro^255^ was added and the ω angle was rotated from the initial angle (ω = −174°, *trans*) to the angle corresponding to *cis* form (ω = 0°) with clockwise or counterclockwise direction, as seen from the N-terminal to the C-terminal direction along the C–N bond, at a rate of 0.2° per 2 ps (Fig. 4*A*). The simulation identified that the distance between the H^N^ atom of TF^PPD^ Ile^195^ and the carbonyl oxygen of MBP Gly^254^ (H^N^–O distance) decreased as the ω angle rotates in clockwise direction. At ∼0.8 ns, the ω angle reached approximately to −90° (*syn* state), and the distance became within the range of the formation of a strong hydrogen bond (< 2.5 Å) (Fig. 4*B*). For example, at 0.200 ns of the clockwise rotation, where the ω angle was −160° (*trans* state), the H^N^–O distance was 4.3 Å, and the angle between the N and H^N^ atoms of TF^PPD^ Ile^195^ and the carbonyl oxygen of MBP Gly^254^ (N–H^N^–O angle) was 134° (Fig. 4*C*). Thus no formation of the intermolecular hydrogen bond between TF^PPD^ Ile^195^ and MBP Gly^254^ was detected at the *trans* state. On the other hand, at 0.794 ns of the clockwise rotation, where the ω angle was −97° (*syn* state), the H^N^–O distance was 2.1 Å, and the N–H^N^–O angle was 152° (Fig. 4*D*). Both the H^N^–O distance and the N–H^N^–O angle at the *syn* state are in the range expected for a strong hydrogen-bond formation (25). It is worth mentioning that the rotation of the peptide bond in the other direction resulted in no shortening of the H^N^–O distance (Fig. 4*B*) and thereby no formation of the intermolecular hydrogen bond.

The simulation also indicated that the intermolecular hydrogen bond formed at the *syn* state tethers MBP Pro^255^ to the hydrophobic cleft of TF^PPD^, and consequently, MBP Pro^255^ and the peptide bond between MBP Gly^254^ and Pro^255^ are closely packed in the hydrophobic cleft (Fig. 4*D*). When the ω angle rotated beyond the *syn* state, the backbone carbonyl oxygen atom of MBP Gly^254^ moves away from the backbone amide group of TF^PPD^ Ile^195^, resulting in the deformation of the intermolecular hydrogen bond and the subsequent release of the close hydrophobic packing between the peptidyl–prolyl bond and TF^PPD^ (Fig. 4*B*). As expected from the structure of TF^PPD^ in complex with MBP (Fig. 3*C*), the C-terminal segment of the MBP (Pro^255^–Trp^258^) was tightly held by TF^PPD^ during the ω angle rotation, and consequently the N-terminal segment preceding MBP Pro^255^ rotates in concurrence with the rotation of the ω angle. As seen in Fig. 4 (*C* and *D*), the position and orientation of MBP Gly^254^ against TF^PPD^ change as the ω angle rotates.

### The intermolecular hydrogen bond through TF^PPD^ Ile^195^ at the transition state is critical for the PPIase activity

**Figure 5.**
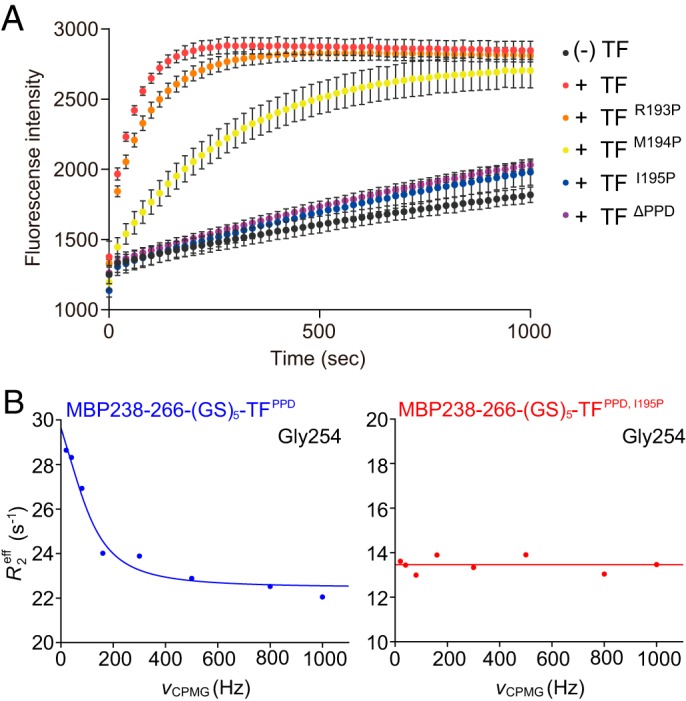
**PPIase activity of TF and TF mutants.** *A*, evaluation of PPIase activity of TF and TF variants by refolding assay of RCM-RNase T1. Refolding of RCM-RNase T1 in the absence and presence of TF or TF mutants was monitored by increase of intrinsic tryptophan fluorescence at 320 nm after excitation at 268 nm. The experiments were performed at 15 °C. Because of the complex process of the refolding of RCM-RNase T1 (37), refolding rates were not extracted. *B*, evaluation of PPIase activity of TF and TF variants by NMR relaxation dispersion experiments. The chemical exchange in MBP Gly^254^ coupled with *cis*/*trans* isomerization of peptidyl–prolyl bond between MBP Gly^254^ and Pro^255^ in complex with TF^PPD^ (*left panel*) or TF^PPD, I195P^ (*right panel*) was monitored.

The role of the intermolecular hydrogen bond between the H^N^ atom of TF^PPD^ Ile^195^ and the carbonyl oxygen of MBP Gly^254^ in the *cis*/*trans* isomerization was evaluated by the activity assays performed using TF mutants, for which mutations of I195P, R193P, and M194P were designed. TF I195P is devoid of the H^N^ atom at the position of 195, and R193P and M194P were designed to perturb the position of the H^N^ atom of TF^PPD^ Ile^195^ as a result of the restricted backbone dihedral angle of the proline residue. To monitor the PPIase activity of the TF mutants, we performed an RNase T1 refolding assay (15), in which the refolding of the reduced and carboxymethylated RNase T1 (RCM-RNase T1) was monitored by the increase of the intrinsic tryptophan fluorescence intensity (Fig. 5*A*). Refolding of RCM-RNase T1 is limited by the slow *trans*-to-*cis* isomerization of peptidyl–prolyl bonds at Pro^39^ and Pro^55^ (15). Although the refolding of RCM-RNase T1 in the absence of TF was slow, it was significantly accelerated by the addition of TF (Fig. 5*A*). However, no enhancement of the refolding was observed in the presence of TF^ΔPPD^, which confirmed that the PPIase activity dominates the foldase activity of TF in the refolding of RCM-RNase T1. The mutants TF^I195P^, TF^M194P^, and TF^R193P^ exhibited significantly reduced activity (Fig. 5*A*). The foldase activity of TF in the refolding of RCM-RNase T1 was more significantly affected when the proline substitution was closer to TF Ile^195^. On the other hand, the mutations of M194A or I195L induced smaller effect in the refolding of RCM-RNase T1 (Fig. S7*A*), supporting the idea that the backbone H^N^ atom of TF^PPD^ Ile^195^ plays an important role in the *cis*/*trans* isomerization. A mutation introduced at His^222^, which forms a hydrogen bond with backbone carbonyl oxygen of MBP Pro^255^, moderately reduced the refolding rate of RCM-RNase T1 (Fig. S7*A*), which suggests that the hydrogen bond mediated by His^222^ is also important in holding the substrate.

High sequence conservation of Ile^195^ and His^222^ in the TF family also supports the importance of the two amino acid residues for PPIase activity (Fig. S8*A*). The comparison of the structures of TF^PPD^ and FKBP12 revealed that TF Ile^195^ and His^222^ are also structurally conserved in FKBP, which suggests a shared isomerization mechanism between these two proteins (Fig. S8*B*).

Note that all of the TF mutants were expressed and purified in a soluble form. Furthermore, ^1^H-^15^N HSQC spectra of TF^PPD-SBD, M194P^ and TF^PPD-SBD, I195P^ clearly showed that the native fold of TF^PPD-SBD^ is preserved even after the introduction of these mutations (Fig. S9, *A* and *B*). The NMR spectra of ^15^N TF^PPD-SBD, I195P^ in the presence of MBP 198–265 indicated that the same binding site on TF^PPD^ is used for the recognition of MBP, which supports the preservation of the interaction between TF^PPD^ and MBP in the TF^I195P^ mutant (Fig. S9*C*). Thus TF^I195P^ binds to the substrate protein but is inactive in the *cis*/*trans* isomerization because of the lack of the ability to form the intermolecular hydrogen bond at the transition state.

The effect of the mutation of I195P was also evaluated by NMR relaxation experiments. We performed ^15^N relaxation dispersion experiments (26, 27) on the fusion proteins: MBP238–266-(GS)_5_-TF^PPD^ and MBP238–266-(GS)_5_-TF^PPD, I195P^(Fig. 5*B*). The backbone amide resonance from MBP Gly^254^ exhibited significant dispersion curve with an exchange rate constant, *k*_ex_, of 740 s^−1^ at 35 °C (Fig. 5*B*, *left panel*), whereas the chemical exchange was disappeared by the introduction of the I195P mutation to TF^PPD^ (Fig. 5*B*, *right panel*). Although a limited number of the resonances were eligible for reliable relaxation dispersion analysis, because of the local process of the peptidyl–prolyl isomerization, as well as the resonance overlap especially for those from MBP peptide, the same trend was seen for a few other resonances around the active site (Fig. S7*B*). Combined with the fact that the MBP Gly^254^ bound to TF^PPD^ indicated only a single set of the resonances whose chemical shifts are in the range expected for *trans* form (Fig. S6*D*), the data indicate that the relaxation dispersion curve seen from the resonance of MBP Gly^254^ reflects the exchange between *trans* and *cis* forms of MBP Pro^255^ as major and minor states, respectively. Note that the exchange for binding and release of MBP is expected to be much faster, given the fact that the binding and release of unfolded PhoA gives rise to *k*_ex_ of ∼1300 s^−1^ at 22 °C (21). The exchange rate indicates that the *cis*/*trans* isomerization of MBP Pro^255^ on TF^PPD^ is quite fast compared with the uncatalyzed isomerization (∼0.01 s^−1^ at 35 °C) (28). Suppression of the chemical exchange by the introduction of the I195P mutation further corroborates the idea that Ile^195^ H^N^ is a catalytic center for the peptidyl–prolyl *cis*/*trans* isomerization by TF^PPD^.

## Discussion

**Figure 6.**
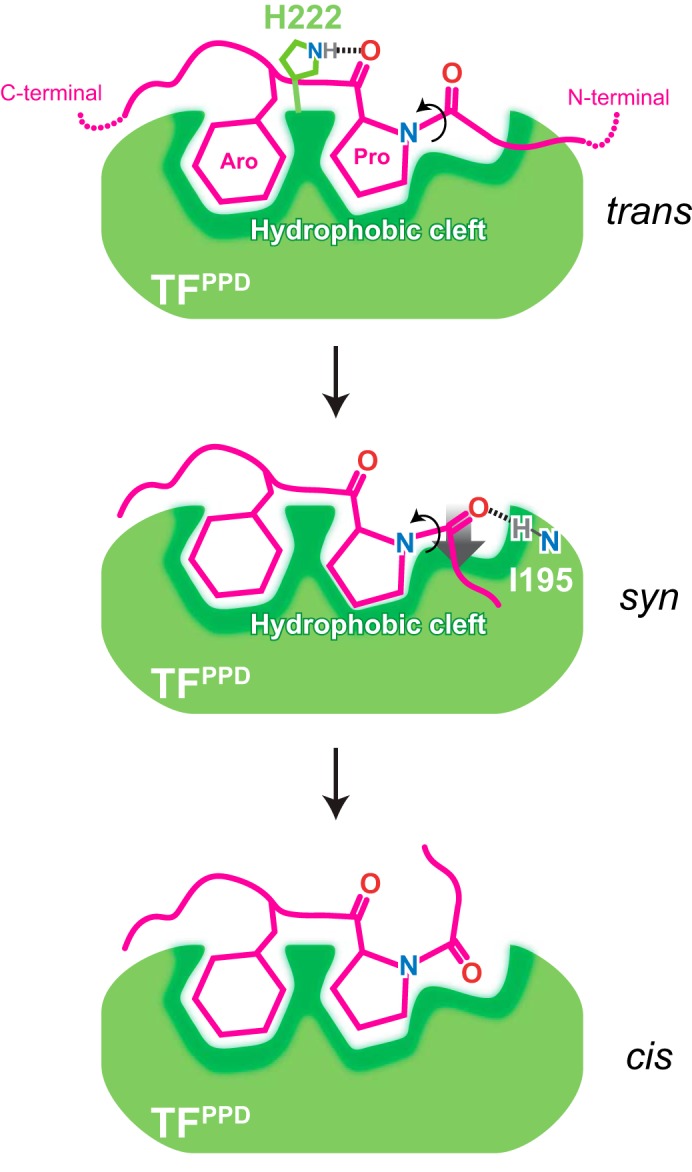
**Schematic representation of peptidyl–prolyl *cis*/*trans* isomerization by TF^PPD^.** TF^PPD^ and the substrate protein are show in *green* and *magenta*, respectively. TF^PPD^ captures the proline-aromatic motif in the *trans* form located in the hydrophobic stretches of the substrate protein. The interaction is mainly mediated by hydrophobic interactions with the conserved hydrophobic cleft of TF^PPD^ that is decollated by TF His^222^ forming a hydrogen bond with the backbone carbonyl oxygen of the proline residue in the substrate. When the peptidyl–prolyl bond rotates to *syn* form, the carbonyl oxygen of the amino acid residue preceding the proline residue forms an intermolecular hydrogen bond with backbone amide group of TF^PPD^ Ile^195^, which tethers the peptidyl–prolyl bond onto the hydrophobic surface of the TF^PPD^. Both the intermolecular hydrogen bond and the hydrophobic environment are important for efficient *cis*/*trans* isomerization. When the peptidyl–prolyl bond rotates to *cis* form, the intermolecular hydrogen bond, and consequently the close hydrophobic contact are released.

The structure corroborated by NMR relaxation analysis, molecular dynamics simulation, and mutational studies led us to propose a mechanistic model of proline *cis*/*trans* isomerization by TF as follows (Fig. 6). As seen in the structure of TF^PPD^ in complex with the unfolded MBP, TF^PPD^ recognizes the proline-aromatic motif located in the hydrophobic stretch of the substrate protein (Fig. 1 and Fig. S6*B*). TF^PPD^ captures the proline-containing peptide in *trans* conformation, using its conserved hydrophobic cleft as decollated by TF His^222^ forming a hydrogen bond with the backbone carbonyl oxygen of the proline residue in the substrate protein (Fig. 3 and Fig. S6, *A* and *D*). Consequently, the proline residue and the C-terminal stretch of the substrate protein are tightly held on TF^PPD^, whereas the residue preceding the proline residue has no significant contact with TF^PPD^ (Fig. 3*C*). However, the MD simulation has revealed that as the peptidyl–prolyl bond rotates, the backbone carbonyl oxygen atom of the amino acid residue preceding the proline residue in the substrate protein moves toward the TF Ile^195^ backbone amide group, which eventually results in the formation of the intermolecular hydrogen bond at the *syn* state with an ω angle of approximately −90° (Fig. 4*D*). Thus the energy barrier of *cis*/*trans* isomerization (∼20 kcal/mol) is expected to be partially compensated by the formation of the intermolecular hydrogen bond with a bond energy of ∼5 kcal/mol (29). Note that the N-terminal stretch of the substrate protein rotates during the isomerization, whereas the proline residue and the C-terminal stretch stay on TF^PPD^ (Fig. 4). The important role of the intermolecular hydrogen bond was further supported by NMR relaxation studies and activity assays in which the perturbations to the backbone amide group of TF Ile^195^ by mutagenesis significantly reduced the PPIase activity of TF (Fig. 5 and Fig. S7*B*). Furthermore, our structural study has also revealed the hydrophobic environment around the peptidyl–prolyl bond. The proline residue in the substrate protein is lodged in the hydrophobic cleft of TF^PPD^ at the ground state (Fig. 3, *B* and *C*), and the formation of the intermolecular hydrogen bond at the transition state tethers the peptidyl–prolyl bond even closer to the hydrophobic surface of the cleft (Fig. 4*D*). Given the fact that the hydrophobic environment promotes *cis*/*trans* isomerization as shown by previous mutational studies (30, 31), our observation suggests that the hydrophobic environment around the peptidyl–prolyl bond at the *syn* state contributes to the isomerase activity of TF^PPD^. Thus we conclude here that the combination of the intermolecular hydrogen bond mediated by TF^PPD^ Ile^195^ H^N^ and the hydrophobic environment around the peptidyl–prolyl bond during the transition is important for eliminating the energy barrier, thereby accelerating the *cis*/*trans* isomerization.

In line with our model, formation of an intermolecular hydrogen bond at the transition state has been reported for cyclophilin A (32). The rotation of the N-terminal segment was also reported for *cis*/*trans* isomerization by cyclophilin A (33, 34). On the other hand, a crystallographic study performed on SlyD suggested that the *cis*/*trans* isomerization is achieved by rotation of the C-terminal segment, *i.e.* the proline residue rotates against the N-terminal segment of the peptidyl–prolyl bond (19). This model was established mainly on the basis of the comparison between the structure of SlyD in complex with the substrate peptides and the immunosuppressant FK506. FK506 contains a pipecolinyl ring that is believed to mimic the proline residue in the twisted transition state (19, 35, 36). Although both TF and SlyD are classified into the FKBP-type PPIase family, SlyD may have a distinct mechanism because of its unique domain architecture consisting of an insert-in-flap chaperone domain.

Our NMR study has also unveiled that the proline residue of MBP in complex with TF^PPD^ is in the *trans* form as a major state (Fig. 3*C* and Fig. S6*D*) and in exchange with *cis* form as a minor state (Fig. 5*B* and Fig. S7*B*). The *cis*/*trans* exchange of the peptidyl–prolyl bond on TF^PPD^ occurs at a rate of 740 s^−1^ at 35 °C (Fig. 5*B* and Fig. S7*B*), which is much faster than the uncatalyzed *cis*/*trans* isomerization of the peptidyl–prolyl bond (∼0.01 s^−1^ at 35 °C) (28, 37). The highly efficient *cis*/*trans* isomerization activity of TF^PPD^ is thereby demonstrated. Although the affinity between isolated TF^PPD^ and the substrate protein is relatively weak, the other two domains of TF, TF^RBD^ and TF^SBD^, may compensate this low affinity. TF^SBD^ contains four of the five substrate-binding sites and binds to the multiple hydrophobic stretches in the substrate protein (21), thereby tethering the substrate protein to TF^PPD^. The previous study indeed has shown that SBD is required for efficient RNase T1 refolding (38). TF^RBD^ binds to the ribosome (39) and consequently tethers TF^PPD^ to the substrate protein emerging out of the ribosome. These features enable TF to exert its activity for broad range of the substrate proteins. Although TF exhibits promiscuous interaction with the unfolded proteins (21, 22) and indeed interacts with most of the newly synthesized proteins emerging out of the ribosome (40), our study has identified that TF^PPD^ possesses the moderate specificity toward the proline-aromatic motif in the hydrophobic stretches of the substrate protein (Fig. 1 and Fig. S6*B*). This moderate specificity of TF^PPD^ suggests that TF^PPD^ preferentially accelerates the proline *cis*/*trans* isomerization of the peptidyl–prolyl bond in the hydrophobic stretch of the substrate protein that is eventually folded into the core of the protein in its native fold. Collectively, the moderate sequence specificity toward proline-aromatic motif in the hydrophobic stretch, as well as the relatively weak affinity to the substrate protein compensated by the other two domains of TF, can be important properties of PPIase embedded in a general chaperone as a functional module.

## Experimental procedures

### Expression and purification of protein samples

TF from *Escherichia coli* was cloned into the pCold vector (Takara Bio). The following TF expression constructs were prepared: TF^SBD^ (residues 113–432Δ150–246) was cloned into pET16b vector (Novagen) and fused to His_6_-MBP and a tobacco etch virus (TEV) protease cleavage site at the N terminus. TF^PPD^ (residues 148–249) and TF^PPD-SBD^ (residues 113–246) were cloned into pCold vector (Takara Bio) and fused to His_6_ tag. TF mutants were constructed by site-directed mutagenesis using a PrimeSTAR mutagenesis basal kit (Takara Bio). The constructs of TF^PPD^, TF^PPD-SBD^, and TF having mutations of R193P, M194P, M194A, I195P, I195L, or H222A were prepared. A fusion protein MBP238–266-(GS)_5_-TF^PPD^ was cloned into pET-16b vector containing a His_6_-MBP and a TEV protease cleavage site at the N terminus. The length of the GS linker was designed from the following consideration. The crystal structure of TF (PDB code 1W26) shows that the N terminus of TF^PPD^ is located ∼25 Å away from the substrate-binding site. More specifically, the distance between C_α_ atoms of Q148 (N terminus of TF^PPD^) and Ile^195^, which is located at the center of the expected binding site, is 26 Å. Given the fact that the length of the fully extended 10-amino acid polypeptide chain is estimated as 35 Å, the five repeats of Gly-Ser should provide sufficient length to preserve the interaction between the two isolated proteins.

All of the expression constructs were transformed into BL21(DE3) cells. The following MBP fragments were prepared in this study: MBP29–99, MBP97–164, MBP160–201, MBP198–265, MBP260–336, and MBP331–396. The MBP fragments were cloned into the pET-16b vector containing a His_6_-MBP and a TEV protease cleavage site at the N terminus. For the unlabeled samples, the cells were grown in Luria–Bertani medium at 37 °C in the presence of ampicillin (100 μg ml^−1^). Protein expression was induced by the addition of 0.2–0.5 mm isopropyl-β-d-1-thiogalactopyranoside at *A*_600_ of ∼0.6, followed by 12–16 h of incubation at 18 °C. The cells were harvested at *A*_600_ of ∼2.0 and resuspended in lysis buffer containing 50 mm Tris-HCl (pH 8.0), 500 mm NaCl. Isotopically labeled samples for NMR studies were prepared by growing the cells in minimal (M9) medium. The cells were harvested at *A*_600_ of ∼1.0. U-^13^C,^15^N-labeled samples were prepared by supplementing the medium with ^15^NH_4_Cl (1 g liter^−1^) and ^13^C_6_-glucose (2 g liter^−1^). The cells were disrupted by sonicator and centrifuged at 18,000 rpm for 45 min. Proteins were purified using nickel-Sepharose 6 Fast Flow resin (GE Healthcare), followed by tag removal by TEV protease at 4 °C (incubation for 16 h) and gel filtration using Superdex 75 16/60 (GE Healthcare). Protein concentration was determined spectrophotometrically at 280 nm using the corresponding extinction coefficient.

### NMR spectroscopy

NMR samples are prepared in 20 mm potassium phosphate buffer (pH 7.0), 100 mm KCl, 4 mm β-mercaptoethanol, 0.5 mm EDTA, 0.05% NaN_3_, and 7% D_2_O. The protein concentration was 0.1–0.8 mm. NMR experiments were performed on Agilent UNITY Inova 800 and 600 MHz NMR spectrometers, as well as Bruker Avance III 800 and 600 MHz NMR spectrometers. The experiments were performed at 10 °C for isolated MBP fragments and at 22 °C for the other samples. The spectra were processed using the NMRPipe program (41), and data analysis was performed with Olivia (https://github.com/yokochi47/Olivia).^4^ The chemical shift changes of the amide moiety were normalized according to the following equation.

(Eq. 1) Δδ=(Δδ(H1))2+(Δδ(N15)/5)2

### Structure determination

To increase the population of the bound state and thus to obtain sufficient number of intermolecular NOEs for high resolution structure determination, a fusion protein (42) in which the peptide containing the binding site (MBP238–266) was fused to the N terminus of TF^PPD^ with a linker consisting of 5 repeat units of Gly-Ser was constructed and used in the NOE observation. Two- and three-dimensional NMR experiments were carried out using Agilent UNITY Inova 800 and 600 MHz NMR spectrometers for the NMR sample containing 1.0 mm ^13^C/^15^N-labeled MBP238–266-(GS)_5_-TF^PPD^ in 20 mm potassium phosphate buffer (pH 7.0), 100 mm KCl, 4 mm β-mercaptoethanol, 0.5 mm EDTA, 0.05% NaN_3_, and 7% D_2_O. The ^1^H, ^13^C, and ^15^N resonance assignments were carried out using the following set of the spectra measured at 22 °C; ^1^H-^15^N HSQC, ^1^H-^13^C HSQC, HNCO, HNCA, HN(CO)CA, HNCACB, CBCA(CO)NH, HN(CA)HA, HBHA(CO)NH, CCH-TOCSY, HC(C)H-TOCSY, HBCBCGCDHD, and HBCBCGCDCEHE. The ^1^H, ^13^C, and ^15^N chemical shifts were referred to DSS (4,4-dimethyl-4-silapentane-1-sulfonic acid) according to the International Union of Pure and Applied Chemistry recommendation. Interproton distance restraints for structural calculations were obtained from ^13^C-edited NOESY–HSQC and ^15^N-edited NOESY–HSQC spectra with a 100-ms mixing time at 22 °C.

The structure was calculated using the CYANA software package (43), on the basis of the interproton distance restraints from the NOESY spectra. The NOE restraints were further corroborated by the dihedral angle restraints from the TALOS+ program (44) and hydrogen-bond restraints for the regions forming secondary structures. 100 structures were calculated individually using 10,000 steps of simulated annealing, and a final ensemble of 20 structures was selected based on CYANA target function values. The 20 lowest-energy structures resulted from CYANA calculation were refined by restrained molecular dynamics in explicit water with CNS (45). The atomic coordinates and structural restraints of MBP238–266-(GS)_5_-TF^PPD^ have been deposited in the Protein Data Bank (PDB code 5ZR0).

### ^15^N relaxation dispersion experiment

An ^15^N-Carr–Purcell–Meiboom–Gill (CPMG) pulse sequence (46) was used to examine the chemical exchange derived from *cis*/*trans* isomerization of Pro^255^ in MBP238–266-(GS)_5_-TF^PPD^ or MBP238–266-(GS)_5_-TF^PPD, I195P^. Two-dimensional data sets were acquired as 240 × 2048 complex points in the *t*_1_ × *t*_2_ time-domain dimensions with a constant relaxation delay of 50 ms. The experiments were performed on Bruker Avance III 800 MHz NMR spectrometers at 35 °C. The exchange rate constant for the two conformers, *k*_ex_, was extracted using NESSY (47) with the Meiboom equation (48) as below, which was applied to the fast exchange processes between the two states,

(Eq. 2) $$R_{2}^{\text{eff}}=R_{2}^{0}+\frac{\varphi }{k_{\text{ex}}}\left[1-\frac{4v_{\text{CPMG}}}{k_{\text{ex}}}\text{tanh}\left(\frac{k_{\text{ex}}}{4v_{\text{CPMG}}}\right)\right]$$

(Eq. 3) $$\varphi =p_{\text{a}}\times p_{\text{b}}\times \delta \omega^{2}$$

where *R*_2_^eff^ is the effective transverse relaxation rate, *R*_2_^0^ is the effective transverse relaxation rate at infinite ν_CPMG_, *p*_a_ and *p*_b_ are the populations of the two state models (*p*_a_ + *p*_b_ = 1), *k*_ex_ is the chemical/conformational exchange (*k*_ex_ = *k*_a-b_ + *k*_b-a_) constant, and δω is the chemical shift difference between states. With this equation, only *R*_2_^0^, *k*_ex_, and Φ can be extracted, because *p*_a_, *p*_b_, and δω cannot be uniquely determined. Note that the fast exchange regime was judged on the basis of the comparison between the expected δω and the observed *k*_ex_. For example, the ^1^H-^15^N HSQC spectrum for ^15^N MBP 198–265 shows the two sets of the resonance for Gly^254^, corresponding to the *trans* and *cis* conformations of Pro^255^ (Fig. S6*C*). The ^15^N chemical shift difference between the two resonances, ∼0.8 ppm and thus ∼61 Hz on 800 MHz NMR instrument, which corresponds to δω in the relaxation dispersion experiment, is much smaller than the *k*_ex_ value (740 s^−1^).

### Molecular dynamics simulations

The molecular simulations were carried out using the AMBER14 molecular dynamics package and the ff14SB force field parameters (49). The initial structure for the simulations was prepared from the lowest-energy structure of MBP238–266-(GS)_5_-TF^PPD^ determined by NMR. The simulation systems were solvated in a cubic periodic box with TIP3P water molecules. The systems were subjected to 5,000 steps of energy minimization and were equilibrated for 3 ns in an isothermal–isovolumetric (NVT) condition with the solute atoms constrained with the force constant of 1 kcal/mol Å^−2^. Several initial structures were obtained during the constrained MD runs. After the equilibration, the production simulations with constrained were carried out in an isothermal–isobarometric (NPT) condition at 1 atm and 300 K. Simulations were performed using a 2-fs time step, periodic boundary conditions, particle mesh Ewald electrostatics, and constraints of hydrogen-containing bonds using the SHAKE algorithm (50). The position restraints with the force constants of 50 kcal/mol rad^2^ was applied to the dihedral angle varying the center of the angle every 0.2° from initial angle (−174°) to 0° with clockwise and counterclockwise, respectively. For each position, the simulations were carried out for 2 ps, resulting in 1.74- and 1.86-ns trajectories for clockwise and counterclockwise rotations, respectively. Although the peptidyl–prolyl *cis*/*trans* isomerization is a slow process that occurs in millisecond timescale, this slow process is due to the low probability of the transition, and a single transition only takes a few nanoseconds (32), which corresponds to the trajectories in this simulation.

### ITC experiment

Calorimetric titrations were carried out on an iTC200 microcalorimeter (GE Healthcare) at 22 °C. All protein samples were purified in ITC buffer containing 20 mm potassium phosphate buffer (pH 7.0), 100 mm KCl, and 0.05% NaN_3_ by gel filtration. The 200-μl sample cell was filled with 350 μm MBP198–265, and the 40-μl injection syringe was filled with 3.5 mm solution of TF^PPD-SBD^. The titrations were carried out with a preliminary 0.2-μl injection, followed by nine injections of 4.2 μl each with time intervals of 5 min. The solution was stirred at 1000 rpm. Data for the preliminary injection, which are affected by diffusion of the solution from and into the injection syringe during the initial equilibration period, were discarded. Binding isotherms were generated by plotting heats of reaction normalized by the modes of injectant *versus* the ratio of total injectant to total protein per injection. The data were fitted with Origin 7.0 (OriginLab Corporation).

### RNase T1 refolding assay

RNase T1 from *Aspergillus oryzae* (Sigma; R-1003) was denatured, reduced, and carboxymethylated using DTT as the reducing agent according to the previous report (51). Refolding of reduced and carboxymethylated RNase T1 (RCM-RNase T1) in 0.1 m Tris-HCl (pH 8.0), 0.4 m NaCl was initiated by 4-fold rapid dilution into the buffer containing 0.1 m Tris-HCl (pH 8.0), 2.0 m NaCl. The final concentration of RCM-RNase T1 was 1 μm. The refolding process of RCM-RNase T1 in the absence and presence of TF or its variants at the concentration of 0.2 μm was monitored by an increase in tryptophan fluorescence intensity. Fluorescence intensity was measured using a spectrofluorometer (FP-8500; JASCO Corporation). The excitation and emission wavelengths were set at 268 nm (bandwidth 5 nm) and 320 nm (bandwidth 10 nm), respectively. All measurements were performed at 15 °C.

## Author contributions

S. K., H. N., H. K., and T. S. data curation; S. K., H. N., and H. K. formal analysis; S. K., H. N., and T. S. investigation; S. K. and T. S. writing-original draft; S. K., K. I., and T. S. project administration; H. N. software; H. N., K. I., and T. S. writing-review and editing; K. I. and T. S. supervision; T. S. conceptualization; T. S. funding acquisition.

## Supplementary Material

Supporting Information

## Footnotes

4 Please note that the JBC is not responsible for the long-term archiving and maintenance of this site or any other third party hosted site.

## Footnotes

3 The abbreviations used are:

TF: trigger factor
PPIase: peptidyl–prolyl *cis*/*trans* isomerase
PPD: PPIase domain
FKBP: FK506-binding protein
RBD: ribosome-binding domain
SBD: substrate-binding domain
MD: molecular dynamics
MBP: maltose-binding protein
HSQC: heteronuclear single quantum coherence
ITC: isothermal titration calorimetry
RCM-RNase: reduced and carboxymethylated RNase
TEV: tobacco etch virus
PDB: Protein Data Bank
CPMG: Carr–Purcell–Meiboom–Gill.
