# Supplementary material for: Structural insight into proline cis/trans isomerization of unfolded proteins catalyzed by the trigger factor chaperone
Source: J Biol Chem. 2018 Aug 9;293(39):15095–106. doi: 10.1074/jbc.RA118.003579 (PMC6166725; doi:10.1074/jbc.RA118.003579)
Supplement: Supporting Information [file supp_293_39_15095__index.html]

Structural insight into proline cis/trans isomerization of unfolded proteins catalyzed by the Trigger Factor chaperone — Proline isomerization by TF chaperone in protein folding — Structural insight into proline cis/trans isomerization of unfolded proteins catalyzed by the trigger factor chaperone — Proline isomerization by TF chaperone in protein folding — Supporting Information 

# Structural insight into proline *cis*/*trans* isomerization of unfolded proteins catalyzed by the trigger factor chaperone

## Supporting Information

- Supporting Information (to be published online) - Figure S1. Identification of TF-binding sites in MBP by NMR. Figure S2. Amino acid composition of TF-binding sites in MBP. Figure S3. Thermodynamic characterization of the interaction between TF and MBP. Figure S4. Investigation of the interaction between MBP and TFSBD or TFPPD by NMR. Figure S5. Evaluation of MBP238-266-(GS)5-TFPPD fusion by NMR. Figure S6. Recognition of MBP by TFPPD. Figure S7. Evaluation of the PPIase activity of TF and TF mutants. Figure S8. Conservation of the amino acid residues of TFPPD involved in the cis/trans isomerization. Figure S9. Mutants of TFPPD-SBD characterized by NMR. Table S1. Structural and NMR statistics of TFPPD-MBP complex.
