# Supplementary material for: Structural insight into proline cis/trans isomerization of unfolded proteins catalyzed by the trigger factor chaperone
Source: J Biol Chem. 2018 Aug 9;293(39):15095–106. doi: 10.1074/jbc.RA118.003579 (PMC6166725; doi:10.1074/jbc.RA118.003579)
Supplement: Supporting Information [file supp_RA118.003579_137595_2_supp_181944_pd6d9x.pdf]

Supporting Information

for

Structural insight into proline *cis/trans* isomerization of unfolded proteins catalyzed by Trigger Factor chaperone

Soichiro Kawagoe, Hiroshi Nakagawa, Hiroyuki Kumeta, Koichiro Ishimori, Tomohide Saio

Figure S1. Identification of TF-binding sites in MBP by NMR.

Figure S2. Amino acid composition of TF-binding sites in MBP.

Figure S3. Thermodynamic characterization of the interaction between TF and MBP.

Figure S4. Investigation of the interaction between MBP and TF^{SBD} or TF^{PPD} by NMR.

Figure S5. Evaluation of MBP238-266-$(GS)_5$-TF_{PPD} fusion by NMR.

Figure S6. Recognition of MBP by TF^{PPD}.

Figure S7. Evaluation of the PPIase activity of TF and TF mutants.

Figure S8. Conservation of the amino acid residues of TF^{PPD} involved in the *cis/trans* isomerization.

Figure S9. Mutants of $TF^{PPD\text{-}SBD}$ characterized by NMR.

Table S1. Structural and NMR statistics of TF^{PPD}-MBP complex.

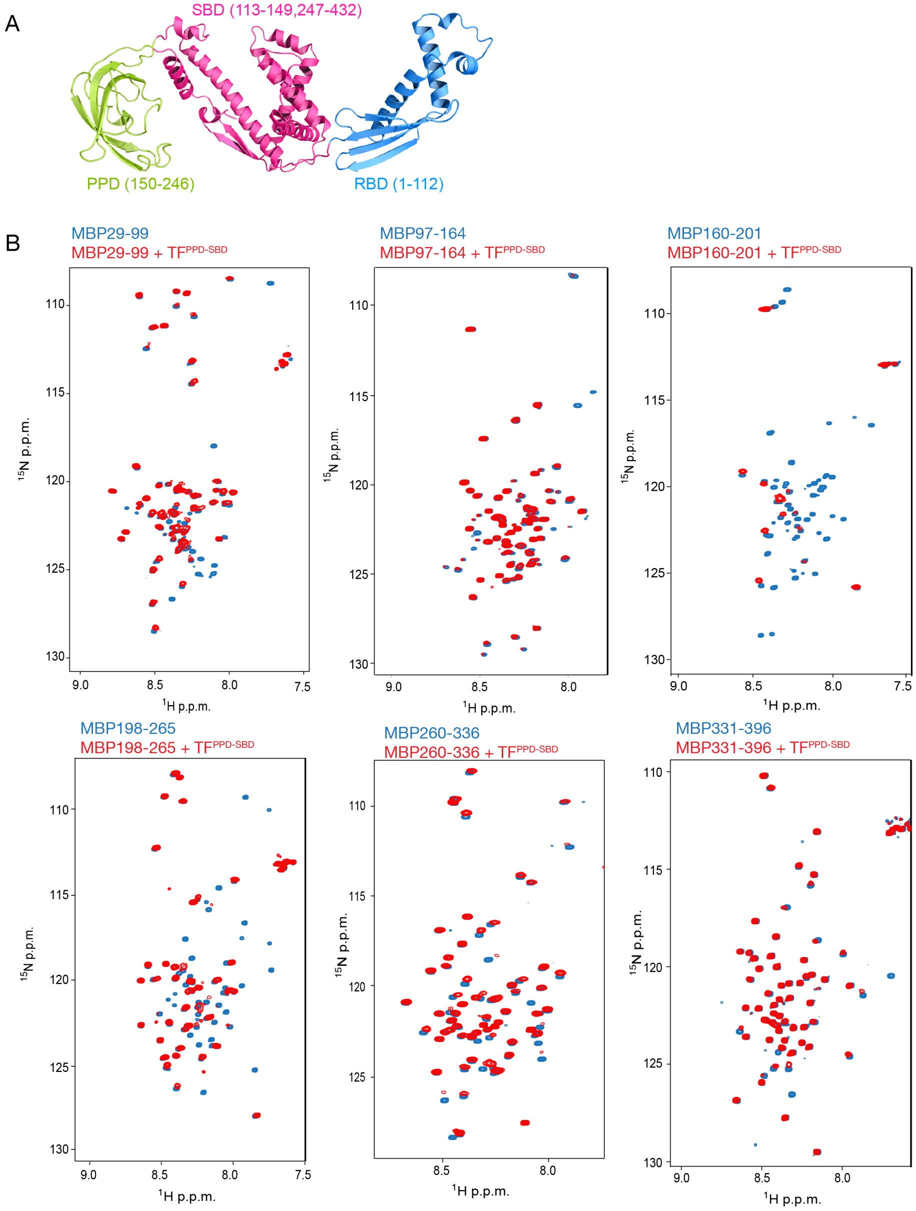

Figure S1. Identification of TF-binding sites in MBP by NMR. (A) Structure of *E. coli* TF (PDB ID: 1W26). PPD, SBD, and RBD are shown in green, pink, and blue, respectively. The residue boundaries for each one of the three domains are shown in parentheses. SBD is discontinuous and is formed primarily by the C-terminal domain. (B) ^{1}H-^{15}N HSQC spectra of MBP fragments in the absence (blue) and presence (red) of TF$^{PPD-SBD}$, having all of the five substrate-binding sites of TF. The fragments of MBP cover the entire region of MBP except the signal sequence. The spectra were recorded for several titration points, MBP:TF 1:0.1, 1:0.2, 1:0.3, 1:0.5, 1:0.7, 1:1, and 1:2, but only the spectra for the ratio of 1:1 were shown here. Concentration of the isotopically labeled MBP fragment was 0.2 mM. The addition of TF$^{PPD-SBD}$ induced significant intensity reduction.

A

```
        10         20         30         40         50         60         70
MKIKTGARIL ALSALTTMMF SASALAKIEE GKLVIWINGD KGYNGLAEVG KKFEKDTGIK VTVEHPDKLE
        80         90        100        110        120        130        140
EKFPQVAATG DGPDIIFWAH DRFGGYAQSG LLAEITPDKA FQDKLYPFTW DAVRYNGKLI AYPIAVEALS
       150        160        170        180        190        200        210
LIYNKDLLPN PPKTWEEIPA LDKELKAKGK SALMFNLQEP YFTWPLIAAD GGYAFKYENG KYDIKDVGVD
       220        230        240        250        260        270        280
NAGAKAGLTF LVDLIKNKHM NADTDYSIAE AAFNKGETAM TINGPWAWSN IDTSKVNYGV TVLPTFKGQP
       290        300        310        320        330        340        350
SKPFVGVLSA GINAASPNKE LAKEFLENYL LTDEGLEAVN KDKPLGAVAL KSYEEELAKD PRIAATMENA
       360        370        380        390
QKGEIMPNIP QMSAFWYAVR TAVINAASGR QTVDEALKDA QTRITK
```

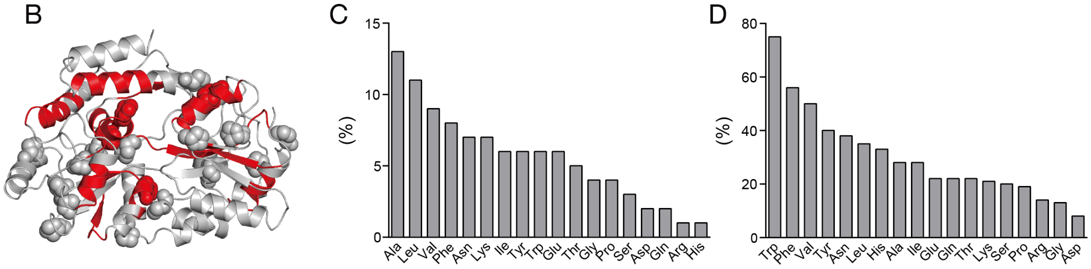

Figure S2. Amino acid composition of TF-binding sites in MBP. (A) Primary sequence of MBP with the TF-binding sites colored red. The signal sequence is colored blue. Proline residues are indicated by underlines. (B) Structure of MBP (PDB ID: 1ANF) with proline residues represented as sphere model. The regions recognized by TF as determined by NMR titration experiments are colored red. (C) Percentage values of the MBP amino acids that interact with TF. (D) Percentage values of the MBP amino acids that interact with TF normalized against the total number of each one of amino acids in the MBP sequence.

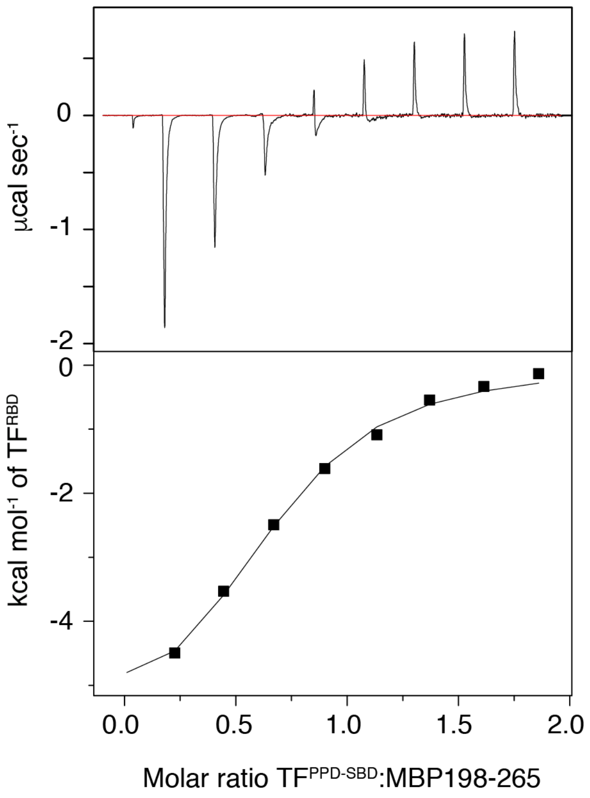

Figure S3. Thermodynamic characterization of the interaction between TF and MBP. ITC traces of the titration of $TF^{PPD\text{-}SBD}$ to MBP198-265 performed at 22 °C, showing that $TF^{PPD\text{-}SBD}$ binds to MBP198-265 with K_d of 47 ± 9 μM and formation of the complex is enthalpy driven.

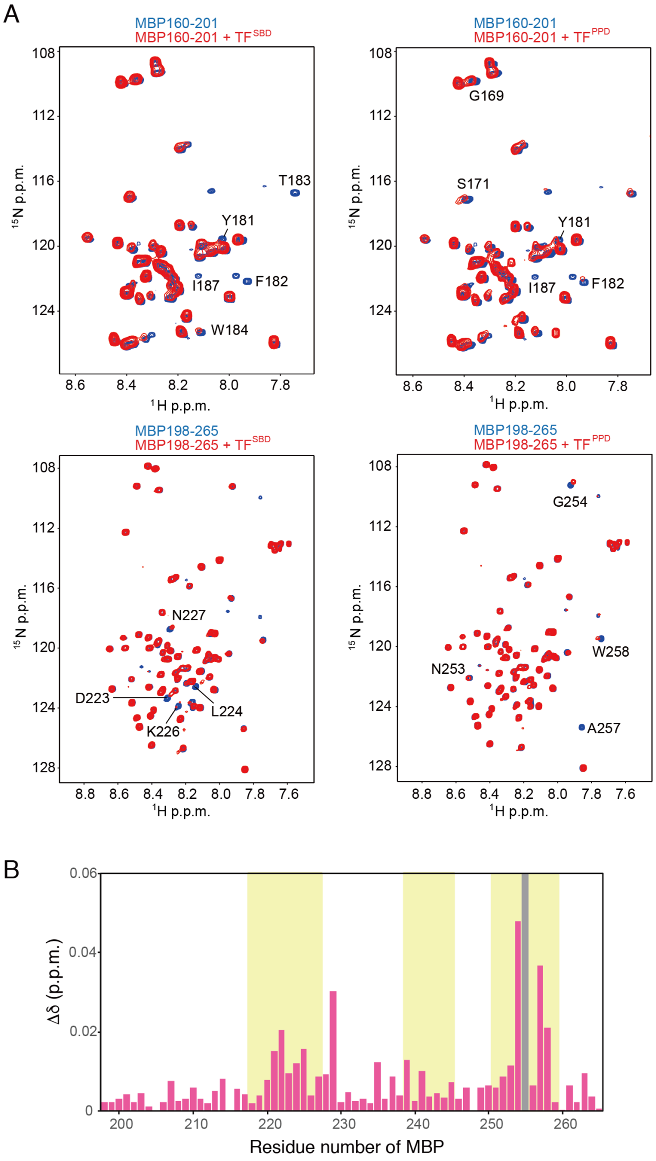

Figure S4. Investigation of the interaction between MBP and TF^{SBD} or TF^{PPD} by NMR. (A) ^{15}N labeled MBP160-201 and MBP198-265 were titrated with unlabeled TF^{SBD} or TF^{PPD}. The ^{1}H-^{15}N HSQC spectra of MBP160-201 and MBP198-265 are shown in the absence (blue) and presence (red) of TF^{SBD} or TF^{PPD}. The spectra were recorded for several titration points, MBP:TF 1:0.1, 1:0.2, 1:0.3, 1:0.5, 1:0.7, 1:1, and 1:2, but only the spectra for the ratio of MBP:TF^{SBD} 1:0.2 and MBP:TF^{PPD} 1:0.5 were shown here. Concentration of the isotopically labeled MBP fragment was 0.2 mM. (B) Plots of chemical shift change of MBP198-265 by the addition of TF^{PPD} at a ratio of MBP:TF^{PPD} 1:0.5. The proline residue, P255, showing no backbone amide resonance on the spectra is indicated by gray bar.

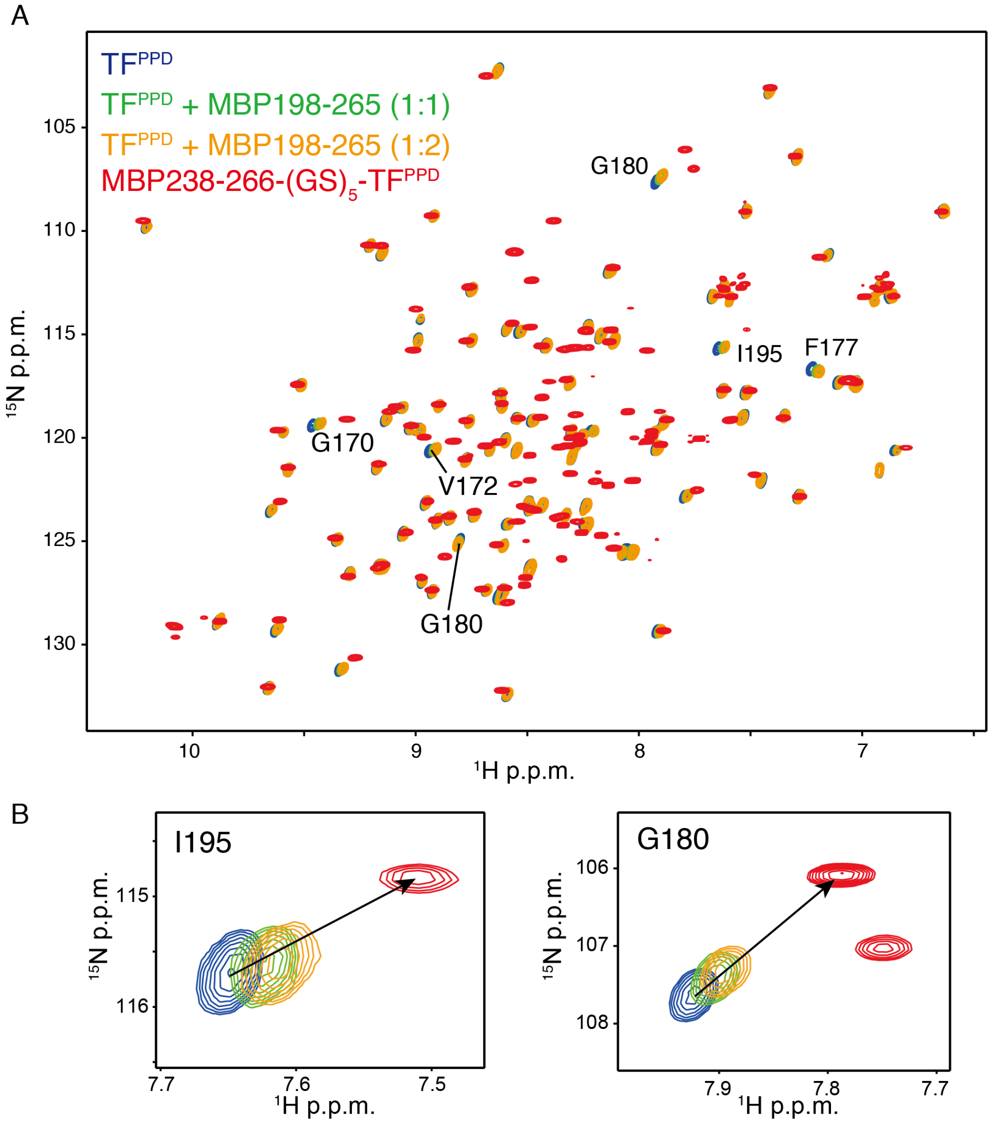

Figure S5. Evaluation of MBP238-266-(GS)$_5$-TF^{PPD} fusion by NMR. (A) ^{1}H-^{15}N HSQC spectra of ^{15}N-labeled TF^{PPD} in the absence (blue) and presence of MBP198-265 at TF^{PPD}:MBP 1:1 (green) or 1:2 (orange) ratios superimposed with the spectrum of ^{15}N-labeled MBP238-266-(GS)$_5$-TF^{PPD} fusion (red). The assignments are indicated for representative perturbed resonances. (B) The expanded views of the selected regions of the spectra showing the resonances from I195 (left panel) and G180 (right panel). The directions of the chemical shift change of the resonances from TF^{PPD} upon the fusion of MBP238-266 coincide with those observed by the addition of MBP198-265, indicating that the binding mode between the MBP fragment and TF in the isolated forms is preserved in the fusion protein of MBP238-266-(GS)$_5$-TF^{PPD}. The direction of the chemical shift change is indicated by an arrow.

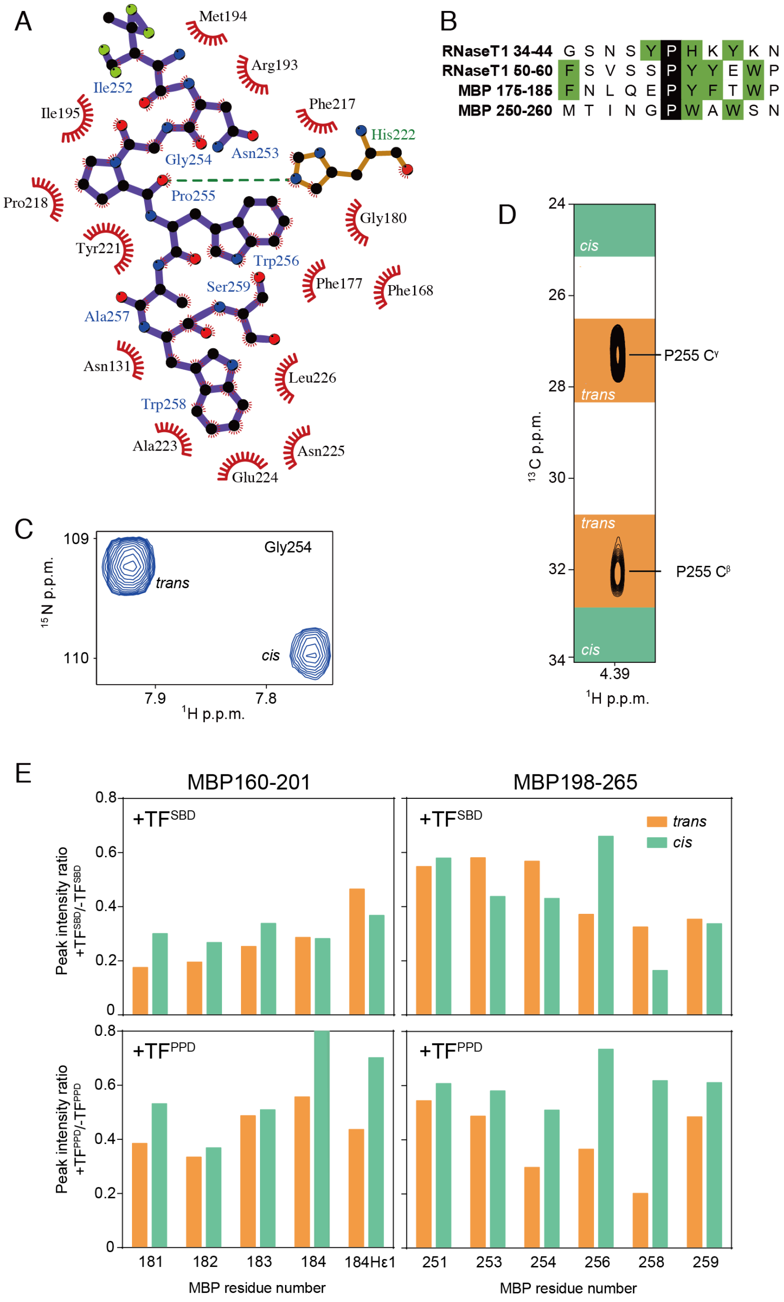

Figure S6. Recognition of MBP by TF^{PPD}. (A) Schematic representation of the intermolecular contacts between TF^{PPD} and MBP generated by Ligplot (http://www.ebi.ac.uk/thornton-srv/software/LIGPLOT/). MBP residues are shown in ball-and-stick representation with purple sticks, and the positions of the residues of TF^{PPD} responsible for the hydrophobic contacts with MBP are indicated by red spoked arcs. The hydrogen bond between TF^{PPD} H222, represented by balls and orange sticks, and MBP P255 is indicated by a green dotted line. (B) Amino acid sequences of the selected regions of RNase T1 and MBP that are recognized

by TF^{PPD}. Proline residues and aromatic residues are highlighted in black and green, respectively. (C) The expanded view of the ^{1}H-^{15}N HSQC spectrum of ^{15}N MBP198-265 showing a set of the resonances from G254 in *trans* and *cis* forms. (D) (H)CCH-TOCSY strip showing the cross peaks for MBP Pro255 C^{β} and C^{γ} atoms in the MBP238-266-$(GS)_5$-TF^{PPD} fusion. The ranges of ^{13}C shifts for *trans* and *cis* isomers (24) are highlighted in orange and green, respectively. (E) Intensity ratio for the resonances from MBP160-201 (left panels) and MBP198-265 (right panels) in the absence and presence of TF^{SBD} (top panels) and TF^{PPD} (bottom panels). The ratios are plotted only for the amino acid residues giving two sets of the resonances corresponding to *cis* and *trans* forms. In both of the MBP fragments, the resonances from *trans* conformation exhibited more significant intensity reduction by the addition of TF^{PPD}, while the trend was not observed by the addition of TF^{SBD}.

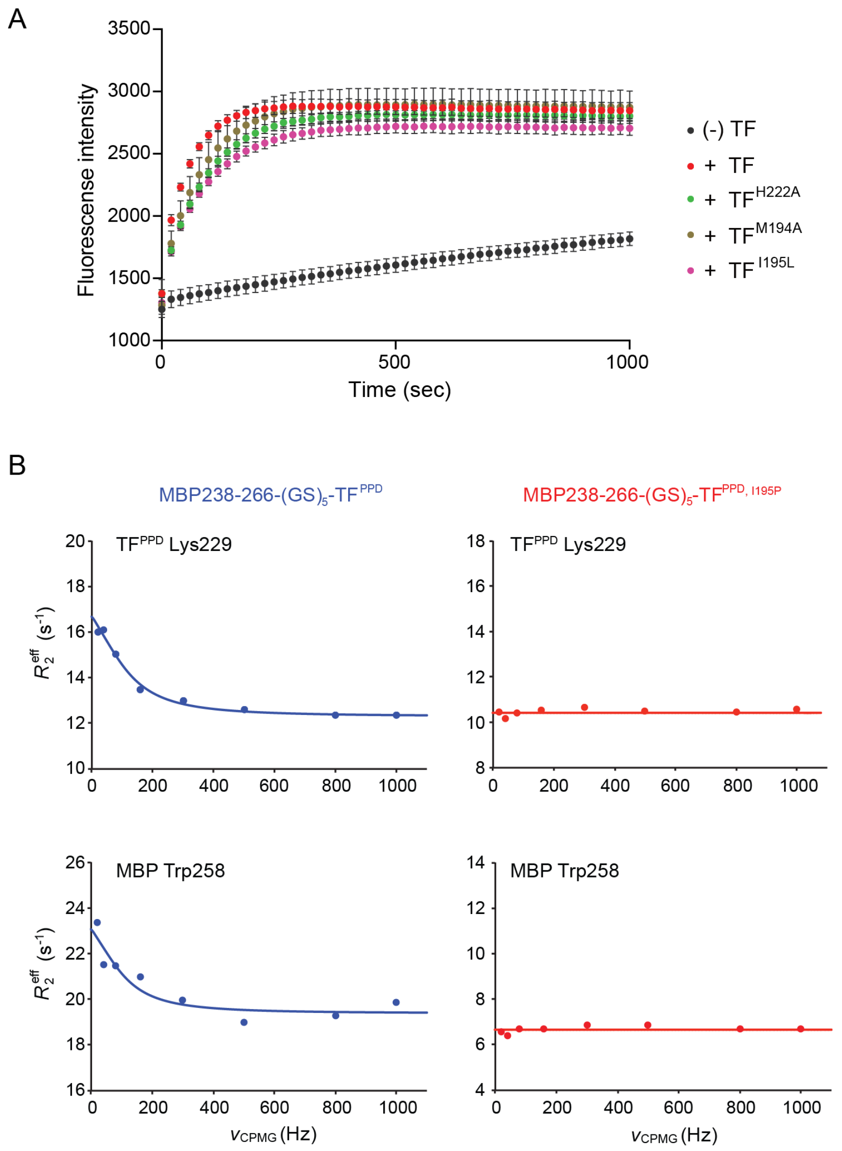

Figure S7. Evaluation of the PPIase activity of TF and TF mutants. (A) Refolding of RCM-RNase T1 in the absence and presence of TF variants. Refolding of RCM-RNase T1 was monitored by increase of intrinsic tryptophan fluorescence at 320 nm after excitation at 268 nm. The experiments were performed at 15°C. (B) NMR relaxation dispersion experiment for MBP238-266-$(GS)_5$-TF^{PPD} (left panels) and MBP238-266-$(GS)_5$-$TF^{PPD,I195P}$ (right panels).

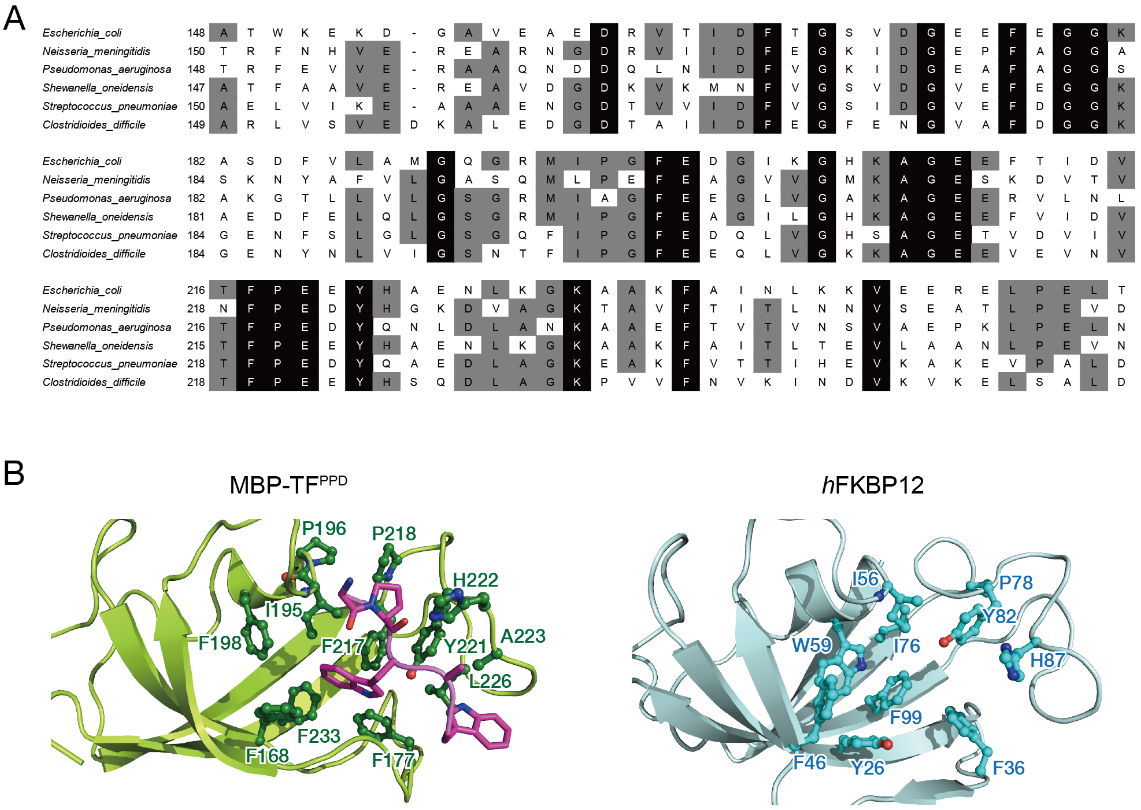

Figure S8. Conservation of the amino acid residues of TF^{PPD} involved in the *cis/trans* isomerization. (A) Multiple sequence alignment for TF^{PPD} from a variety of organisms. The conserved residues are highlighted. (B) Comparison of the structure of TF^{PPD} in complex with MBP (left panel) with that of human FKBP12 (PDB ID: 1FKF) (right panel). TF^{PPD}, MBP, and FKBP12 are colored green, magenta, and cyan, respectively. The residues in the MBP-TF^{PPD} complex that mediates the interaction and the corresponding amino acid residues in FKBP12 are shown in ball-and-stick representation.

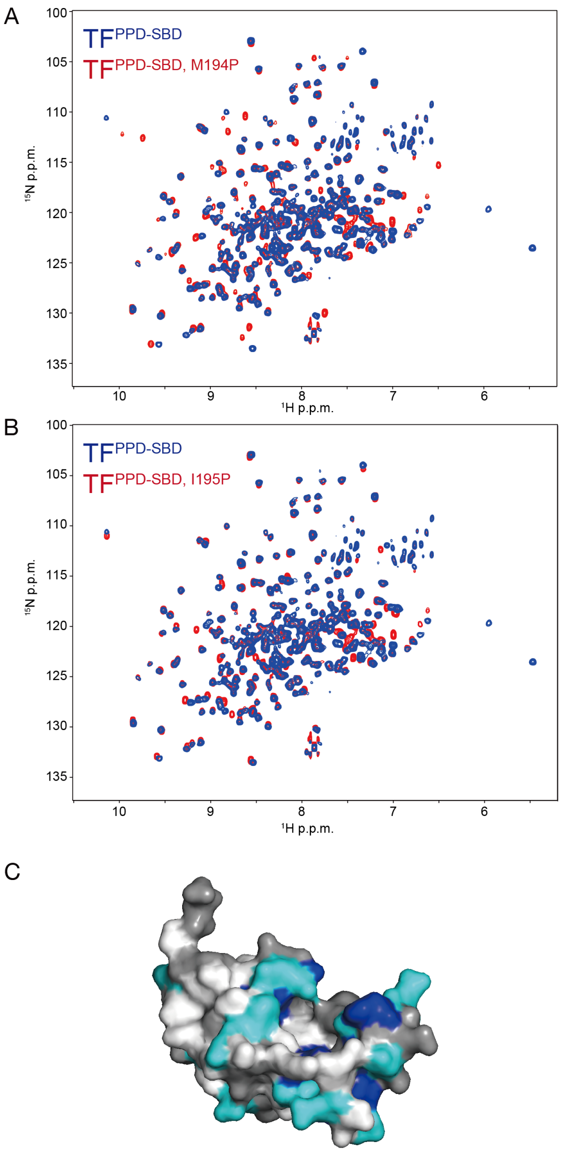

Figure S9. Mutants of TF$^{\text{PPD-SBD}}$ characterized by NMR. Overlay of the ^{1}H-^{15}N HSQC spectra of wild type (blue) and mutants (red) of TF$^{\text{PPD-SBD}}$. The spectra of TF$^{\text{PPD-SBD, M194P}}$ (A) and TF$^{\text{PPD-SBD, I195P}}$ (B) are overlaid with that of wild type TF$^{\text{PPD-SBD}}$. The spectra clearly show that TF$^{\text{PPD-SBD, M194P}}$ and TF$^{\text{PPD-SBD, I195P}}$ maintain the native fold. (C) Mapping of the chemical shift perturbations of TF$^{\text{PPD-SBD, I195P}}$ upon the addition of MBP198-265 at 1:1 ratio. The residues with perturbations larger than the threshold values of 0.015 and 0.025 ppm are colored light blue and dark blue, respectively. Proline and unassigned residues are colored gray.

Table S1. Structural and NMR statistics of TF^{PPD}-MBP complex.

Distance restraints	
NOEs	
Short range (intra-residue and sequential)	598
Medium range (1 < │ i-j │ < 5)	151
Long range (│ i-j │ ≧ 5)	434
Intermolecular	22
Hydrogen bonds	20
Dihedral angle restraints(ϕ and ϕ)	128
Violations (mean and SD)	
Distance restraints(Å)	0.0032 ± 0.01960
Dihedral angle restraints(°)	0.68 ± 1.20
Structural coordinates rmsd[a]	
Backbone atoms	0.76 Å
All heavy atoms	1.21 Å
Ramachandran plot	
Most-favored regions	88.7%
Additionally allowed regions	11.2%
Generously allowed regions	0.1%
Disallowed regions	0.0%

[a]Analysis applied to the residues except the MBP238-266, GS-linker, and the flexible loops.
